# Lingual leiomyomatous hamartoma with bifid tip and ankyloglossia in a patient without oral-facial-digital syndrome: a case report and literature review

**DOI:** 10.1186/1477-7819-11-230

**Published:** 2013-09-16

**Authors:** Hsing-Liang Wang, Feng-Yu Chiang, Chih-Feng Tai, Kun-Bow Tsai, Ling-Feng Wang

**Affiliations:** 1 Department of Otolaryngology-Head and Neck Surgery, Kaohsiung Medical University Hospital, Kaohsiung City, Taiwan; 2 Department of Otolaryngology-Head and Neck Surgery, Faculty of Medicine, College of Medicine, Kaohsiung Medical University, Kaohsiung City, Taiwan; 3 Department of Otolaryngology-Head and Neck Surgery, Kaohsiung Municipal Hsiao-Kang Hospital, Kaohsiung City, Taiwan; 4 Department of Pathology, Kaohsiung Municipal Hsiao-Kang Hospital, Kaohsiung City, Taiwan; 5 Department of Otolaryngology, Kaohsiung Municipal Ta-Tung Hospital, Kaohsiung Medical University, Kaohsiung City, Taiwan

**Keywords:** Lingual leiomyomatous hamartoma, Leiomyomatous hamartoma, Oral-facial-digital syndrome, Tongue tumor, Oral tumor

## Abstract

Here is a rare case of lingual leiomyomatous hamartoma (LLH) with bifid tongue tip and tongue-tie in a patient with non-oral-facial-digital syndrome (OFDS). A 29-year-old male consulted for a painless tumor over the midline of the tongue dorsum measuring 2 × 1.5 cm. The tumor was excised and the tongue-tie was corrected. Diagnosis of LLH was based on histo-pathologic and immuno-histochemical studies. The epidemiologic data and differential diagnosis of LLH, as well as related literature, are discussed. To date, only 14 cases of LLH have been reported in English literature. This may be the first reported case of LLH with bifid tip and ankyloglossia in a non-OFDS patient.

## Background

Hamartoma was first defined by Alberecht [1] in 1904 as a benign, tumor-like malformation composed of a disordered mixture of mature tissues that normally occur in the affected part, but with a predominance of one particular tissue. Hamartomas often occur in the liver, kidneys, lungs, spleen, pancreas, and in the oral region and are classified into many subtypes according to their main component.

Leiomyomatous hamartoma (LH) is composed mainly of smooth muscle tissue intermixed with other adjacent tissue and often occurs in the lungs and kidneys. Cases involving the oral cavity are extremely rare and most have occurred in the Japanese, with few cases among Caucasian and Latin Americans of young age [2,3]. Only 14 cases of LH in the tongue have been reported, most of which are in young children; these have been reported individually or as part of a genetic syndrome, especially oral-facial-digital syndrome (OFDS).

The OFDS is a complex syndrome with 10 different subtypes that include oral, facial, and digital abnormalities. LH with a lobulated tongue tip or short frenulum has been reported in some types of OFDS [4]. This report is of a case of lingual leiomyomatous hamartoma (LLH) with a bifid tip and ankyloglossia in a 29-year-old man without OFDS. The current English literature on LLH is also reviewed.

## Case presentation

**Figure 1:**
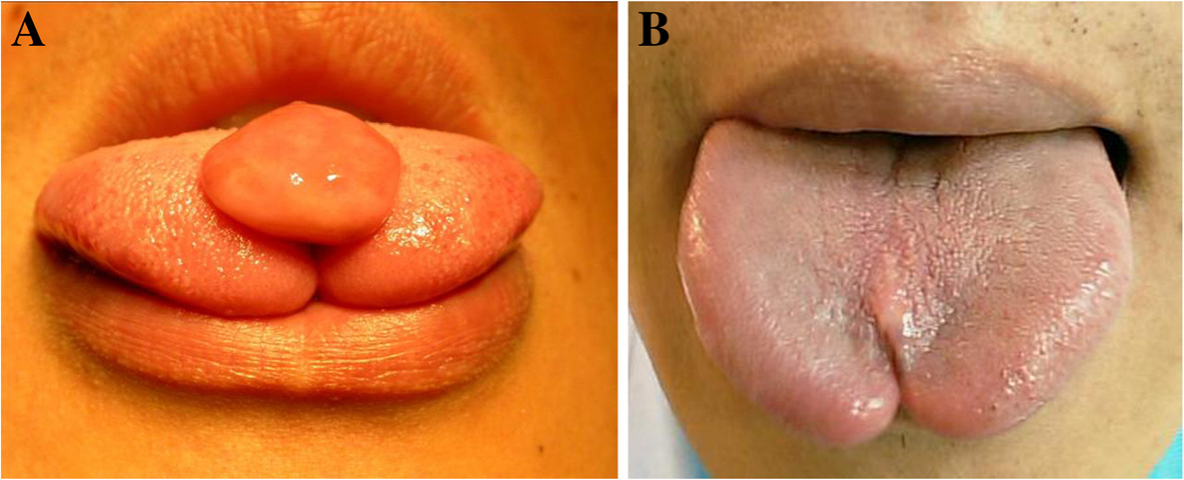
**The preoperative and postoperative appearance of tongue tumor. (A)** Preoperative view of an exophytic, well-defined, reddish, smooth, non-tender mass midline over the tongue dorsum measuring 2 × 1.5 cm in diameter. An M-shaped (bifid) tongue tip and tongue-tie were also noted. **(B)** Four years postoperative the surgical wound was well-healed, with only mild scar formation.

A 29 year-old man was referred in June 2008 for a painless, slow-growing mass over the tongue dorsum that had been noted since childhood. Upon examination, there was evidence of an exophytic, reddish, non-tender mass, measuring 2 × 1.5 cm over the midline of the tongue dorsum. There was also a bifid tongue tip and tongue-tie (Figure 1A). He denied any discomfort associated with this mass. The mass was excised and the tongue-tie was corrected smoothly under general anesthesia.

**Figure 2:**
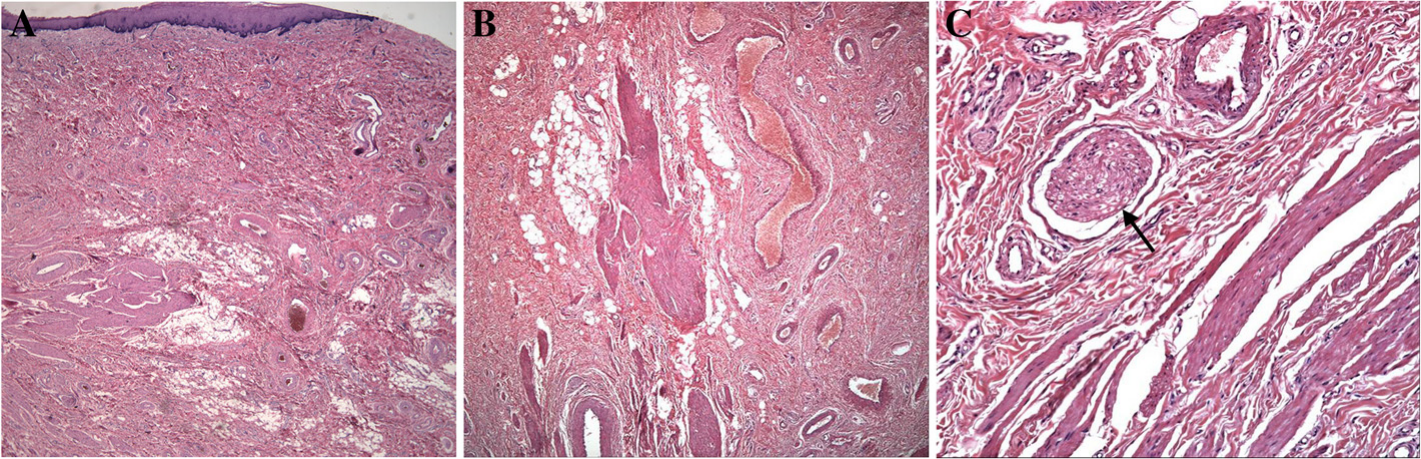
**The histopathological study of LLH in hematoxylin and eosin (H&E) stain. (A)** Muscle bundles, blood vessels, and adipose tissue in the polypoid tumor covered by stratified squamous epithelium (H&E stain; original magnification × 2). **(B)** The non-encapsulated tumor was composed of smooth muscle bundles, blood vessels, and adipose tissue within fibrous stroma (H&E stain; original magnification × 4). **(C)** Smooth muscle bundles admixed with a nerve (arrow) and blood vessels (H&E stain; original magnification × 20).

**Figure 3:**
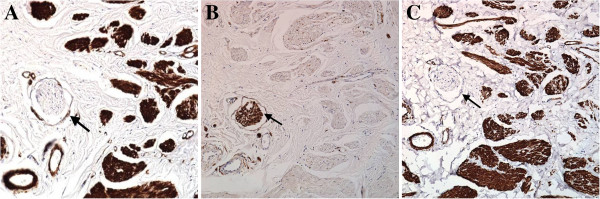
**The histopathological study of LLH in immuno-histochemical stain. (A)** Positive reaction with smooth muscle actin in the muscle bundles, but negative in the nerve (arrow). (Immuno-histochemical stain; original magnification × 20). **(B)** Positive reaction with muscle actin (HHF 35) in the muscle bundles, but negative in the nerve (arrow). (Immuno-histochemical stain; original magnification × 20). **(C)** Positive reaction with S-100 protein in the nerve (arrow), but negative in smooth muscle bundles. (Immuno-histochemical stain; original magnification × 20).

Microscopically, the polypoid tumor was non-encapsulated. It was composed of smooth muscle cells with cigar-shaped nuclei mainly arranged in irregular thick bundles intermingled with blood vessels, nerves, and adipose tissue within fibrous stroma (Figure 2). Immuno-histochemical study (Figure 3) revealed a positive reaction with smooth muscle actin (SMA) and muscle actin (HHF 35) in the smooth muscle bundles and vascular walls. The nerves and adipose tissue were also immunoreactive to the S-100 protein. These features were all characteristic of LH.

The patient was followed up uneventfully for four years after surgical intervention. The surgical wound was well-healed, with mild scar formation (Figure 1B). There was no evidence of recurrence.

## Discussion

This report is the fourteenth known case of lingual LLH but is perhaps the first case of a non-OFDS patient presenting with a bifid tip and ankyloglossia. Of the 14 cases with LLH reported to date (Table 1), ten (71.4%) are male and four (28.6%) are female patients. Most cases have been observed at birth, but age at diagnosis has a range of 8 days to 61 years. The LLH is usually a congenital, self-limited, and slow-growing tumor, such that the patient may not have any discomfort unless the mass suddenly becomes enlarged. In the review of cases, most have been diagnosed at an age <6 years and most lesions are <2 cm in size. Most cases involving the oral region have occurred in the Japanese [5,6], although according to the report of Nava-Villalba [3], 50% of patients with LLH are Caucasian and only three patients are Japanese. To date, the present case may be the first reported Chinese incidence in the world (Table 1).

**Table 1:** Summary of cases of lingual leimyomatous hamartomas reported in English literature

**Case**	**Author**	**Year**	**Ethnicity**	**Age at diagnosis**	**Age at presentation**	**Gender**	**Location**	**Size (mm)**
1	Kanekawa [5]	1990	Japanese	3 yr	Birth	M	Posterior dorsal tongue, upper gingival	-
2	Goldsmith [12]	1995	Caucasian	1yr 4 mo	Birth	M	Posterior dorsal tongue	-
3	Rosa-García [13]	1999	Latin American	6yr	Birth	M	Tongue tip	13 × 3 × 3
4	Kobayashi [2]	2001	Japanese	3 mo	Birth	M	Posterior dorsal tongue	10 × 14 × 5
5	Seiji Iida [8]	2007	Japanese	2 yr 7 mo	4 mo	M	Posterior and anterior dorsal tongue. incisive papilla	4 × 4 × 3
								2
6	Kreiger [9]	2007	Caucasian	8d	Birth	M	Anterior dorsal tongue	1 to 20^a^
7			Caucasian	4mo	Birth	F	Anterior dorsal tongue	
8			Caucasian	5mo	Birth	F	Middle dorsal tongue.	
9			Caucasian	1yr	Birth	M	Anterior dorsal tongue	
10			Caucasian	5yr	-	M	Middle dorsal tongue.	
11	Villalba [3]	2008	Latin American	5mo	Birth	M	Posterior dorsal tongue	7 × 6 × 5
12	De Faria [7]	2008	-	61yr	Birth	F	Posterior tongue	36 × 30 × 20
13	Hahn [14]	2010	Caucasian	2 yr	Birth	F	Midline of tongue base	16 × 13 × 11
14	Present case	2012	Taiwanese	26 yr	Birth	M	Anterior dorsal midline tongue Combined with bifid tongue-tip and ankyloglossia	17 × 15 × 8

^a^Approximate value; M, male; F, female. - : data unavailable.

Kobayashi *et al*. [2] have mentioned that the tumor might become apparent when it enlarges rapidly after recurrent trauma or during the pubertal growth spurt. This might explain the huge LLH (3.6 × 3.0 × 2.0 cm) in De Faria and Batista’ [7] report. Kobayashi *et al*. [2] also mention that oral hamartoma usually arises from the foramen cecum during embryonic development, which may explain why most LLH are in the midline of the tongue. Furthermore, most LLH are solitary and only two cases have multiple lesions [7].

The normal tongue is composed predominantly of skeletal muscle with an overlying, keratinizing, stratified, squamous mucosa. Smooth muscle is part of the normal lingual vasculature but does not occur independently. A hamartoma is a proliferation of normal tissues that are considered endogenous to the site of occurrence. Typically the tissues in hamartomas appear disorganized and ill-defined, merging with the normal surrounding tissues. Within the tongue, endogenous elements that might result in a hamartoma would include vessels, nerves, lymphatics, muscle, fat, and salivary gland tissue. The etiology of the combination of developmental variations: hamartoma + bifid tongue + ankyloglossia (tongue-tie) in our report remains obscure. The sporadic mutation in the transcription factor may occur during the tongue development at the fourth embryonic week, causing the abnormality of morphogenesis at the end stage of the lingual swelling mergence. As poor fusion between the lateral and the medial lingual swelling occurs, the tongue tumor, combined with the bifid tongue and ankyloglossia may occur together on the tip of the tongue midline. Environmental factors, for example, poor nutrition; overuse of vitamins; use of drugs, alcohol or heroin; smoking; and exposure to thalidomide or pathogens may cause sporadic mutation with abnormality of morphogenesis during embryologic development.

In patients with a congenital midline tongue mass, the differential diagnosis includes choristoma, leiomyoma, and benign mesenchymoma. Choristoma [8,9] is a well-organized normal tissue proliferation in abnormal locations, usually occurring in elderly patients. Leiomyoma [10] most commonly arises from the smooth muscle of the female genital tract, uterus, or the esophagus, and rarely occurs in the tongue. Moreover, it is often found in the fourth and fifth decades of life. Benign mesenchymoma [3,7-9] is a circumscribed, unencapsulated benign tumor composed of two or more mesenchymal lineage tissues without any single predominant tissue. No nervous tissues are noted in mesenchymoma [9] even though some may show adjacent tissue infiltration. In LLH, there is disorganized tissue growth and smooth muscle predominance but without local tumor infiltration. These often tend to occur in young patients.

However, there are other rarer tumors that should be put into the differential diagnosis of LLH. First, congenital epulis [4] which usually presents on alveolar gingival mucosa at birth is the most common congenital intra-oral, benign, soft-tissue tumor, and rarely occurs in the dorsal tongue [1]. Second, the congenital genetic syndrome, OFDS, should also be among the differential diagnoses of LLH. The OFDS usually includes malformations of the mouth, teeth, jaw, facial bones, and limbs, together with varying degrees of mental retardation. An LH combined with lobed tongue and mouth frenulum may occur in some types of OFDS. The unusual combination of LLH with bifid tongue tip and ankyloglossia in a non-OFDS patient, as in the present case, has not been previously reported. Third, teratoma is more commonly associated with other dysmorphism than LLH. For example, Andrade and Raikwar [11] mentioned a case of congenital benign teratoma of the tongue with bifid tip, ankyloglossia and polydactyly. The etiology of both hamartoma and teratoma combined with bifid tongue tip and ankyloglossia are still unknown; however, genetic or embryonic events might explain the coincidental finding.

Immunohistochemical characteristics are essential in differentiating soft-tissue neoplasms. The SMA and S-100 protein are the most commonly used markers in confirming the histomorphologic findings in LLH [2-4,7,8]. However, use of vimentin, desmin, and HHF35 has been reported in other articles [2-4,7,8]. SMA is a specific smooth-muscle immunomarker that can be seen in smooth-muscle bundles and vessel walls, in the present case (Figure 3A). The S-100 protein is normally present in cells derived from the neural crest, chondrocytes, adipocytes, myoepithelial cells, macrophages, and keratinocytes. Adipose tissue and nerve fibers may also have positive staining but smooth muscles have negative staining (Figure 3B). So, S-100 protein can be a key immunohistochemical marker to differentiate LLH from vascular or solid leiomyoma. HHF35 reacts with all kind of muscle cells, pericytes, and myoepithelial cells, but is nonreactive with endothelial-, epithelial-, neural-, or connective-tissue cells; this can also confirm the smooth muscle predominant diagnosis in our article (Figure 3C).

Most case reports advocate surgical excision with a safe margin as the treatment of choice for LLH [3,5,9-14]. Prognosis is generally quite good and no post surgical recurrence has been reported in the current literature.

## Conclusions

Here is a special case of LLH combined with bifid tongue tip and tongue-tie in a patient without OFDS. To date, this may be the first such case reported in English literature. It is a reminder that when confronted with a smooth-mass lesion over the midline of the tongue dorsum, leiomyomatous hamartoma should be considered in the differential diagnosis, especially when the patient is a young adult and without OFDS.

## Consent

Written informed consent was obtained from the patient for publication of this case report and any accompanying images (IRB number: KMUH-IRB-20120262). A copy of the written consent is available for review by the Editor-in-Chief of this journal.

## Abbreviations

LLH: Lingual leiomyomatous hamartoma; OFDS: Oral-facial-digital syndrome; SMA: Smooth muscle actin.

## Competing interests

The authors declare that they have no competing interests.

## Authors' contributions

HLW and LFW performed data collection, manuscript composition and submission. CFT and FYC took part in the care of the patient, and performed the literature search. KBT performed pathology analysis and photography. All authors read and approved the final manuscript.
